# Bioconversion of xylose, hexoses and biomass to ethanol by a new isolate of the white rot basidiomycete *Trametes versicolor*

**DOI:** 10.1186/2193-1801-3-121

**Published:** 2014-03-03

**Authors:** Kenji Okamoto, Atsushi Uchii, Ryuichi Kanawaku, Hideshi Yanase

**Affiliations:** Department of Chemistry and Biotechnology, Graduate School of Engineering, Tottori University, 4-101 Koyama, Tottori, 680-8552 Japan

**Keywords:** Xylose, Biomass, Ethanol, Fermentation, White rot basidiomycete

## Abstract

Second-generation bioethanol production requires the development of economically feasible and sustainable processes that use renewable lignocellulosic biomass as a starting material. However, the microbial fermentation of xylose, which is the principal pentose sugar in hemicellulose, is a limiting factor in developing such processes. Here, a strain of the white rot basidiomycete *Trametes versicolor* that was capable of efficiently fermenting xylose was newly isolated and characterized. This strain, designated KT9427, was capable of assimilating and converting xylose to ethanol under anaerobic conditions with a yield of 0.44 g ethanol per 1 g of sugar consumed. In culture medium containing low yeast extract concentrations, xylose consumption and ethanol productivity were enhanced. Adjusting the initial pH between 3.0 and 5.0 did not markedly influence xylose fermentation. *T. versicolor* KT9427 also produced ethanol from glucose, mannose, fructose, cellobiose and maltose at yields ranging from 0.45 to 0.49 g ethanol per 1 g of sugar consumed. In addition, strain KT9427 exhibited favourable conversion of non-pretreated starch, cellulose, xylan, wheat bran and rice straw into ethanol compared to common recombinant yeast strains. Taken together, the present findings suggest that *T. versicolor* KT9427 is a promising candidate for environmentally friendly ethanol production directly from lignocellulosic biomass.

## Background

Lignocellulosic biomass, which is composed of the carbohydrate polymers cellulose, hemicelluloses and lignin, is the most abundant and inexpensive material containing available sugars for bioconversion to ethanol. Bioethanol represents a sustainable and renewable transportation fuel that is a promising and environmentally friendly alternative to gasoline because it reduces the emission of greenhouse gases that contribute to global warming. Currently, nearly all bioethanol produced worldwide is derived from sugar or starchy crops. However, second-generation bioethanol production requires the development of economically feasible and sustainable industrial processes that utilize renewable lignocellulosic materials, including cellulose and hemicellulose, which do not compete with food sources (Hayes 2009).

Consolidated bioprocessing (CBP) using a single microbial species capable of one-step conversion of lignocellulosic biomass to ethanol has received considerable recent attention for cost-effective bioethanol production (Lynd et al. 2005; Xu et al. 2009). For the effective utilization of lignocellulosic biomass, the fermentation of not only hexoses, but also pentose sugars, is critical. In particular, the efficient microbial utilization of xylose, which is the principal pentose sugar of the hemicellulose component of lignocellulosic biomass (Gírio et al. 2010), is required for developing efficient CBP processes. However, the most commonly used yeast species for bioethanol production, *Saccharomyces cerevisiae*, is unable to ferment xylose (Barnett 1976), and only a few species capable of efficiently fermenting this sugar, including *Pachysolen tannophilus*, *Candida* spp., *Kluyveromyces marxianus* and *Pichia stipitis*, have been identified among naturally occurring fungi (Schneider et al. 1981; Jeffries 1981; Margaritis and Bajpai 1982; Gong et al. 1983; du Preez and van der Walt 1983; Toivola et al. 1984). Ascomycetes including *Monilia* sp*.*, *Fusarium oxysporum, Neurospora crassa* and *Paecilomyces lilacinus,* and Zygomycetes including *Mucor indicus* and *Rhizopus oryzae* are also able to convert xylose to ethanol (Gong et al. 1981; Suihko and Enari 1981; Desphande et al. 1986; Mountfort and Rhodes 1991; Millati et al. 2005). As a biocatalyst for the efficient conversion of lignocellulosic biomass into ethanol it is preferable to utilize a microbe that possesses the ability to ferment various types of carbohydrates, which is a characteristic not observed in the above mentioned xylose-fermenting yeasts, ascomycetes and zygomycetes.

Basidiomycetes are considered to be the most capable microorganisms for lignocellulose degradation in nature. A number of white rot basidiomycetes are particularly suited for the biological pretreatment or simultaneous saccharification and fermentation of lignocellulosic biomass (Taniguchi et al. 2005; Bak et al. 2009; Jeya et al. 2009; Shi et al. 2009; Dias et al. 2010). Whereas there are a few studies on white rot basidiomycetes, including *Phanerochaete chrysosporium*, *Flammulina velutipes*, *Peniophora cinerea* and *Trametes suaveolens*, which produce ethanol from hexose sugars (Kenealy and Dietrich 2004; Mizuno et al. 2009; Okamoto et al. 2010), and *T. hirsuta,* which exhibits relatively favourable fermentation of xylose (Okamoto et al. 2011). However, the xylose consumption and ethanol conversion rates of *T. hirsuta* are too slow for industrial applications, as achieving high ethanol productivity from lignocellulosic biomass requires near complete xylose consumption in a short period of time. During the exploration for basidiomycetes with enhanced fermentation abilities, we identified a strain of *T. versicolor* that was capable of efficiently converting not only hexose sugars, but also xylose, to ethanol. Although *T. versicolor* is a well-known white rot basidiomycete with respect to the secretion of ligninolytic enzymes, such as laccases (Bourbonnais and Paice 1992), details of the fermentation system of this basidiomycete remains largely uncharacterized.

The aim of the present study was to characterize the ability of *T. versicolor* to ferment xylose and evaluate the potential of this basidiomycete for efficient bioethanol production by CBP.

## Materials and methods

### Chemicals

All chemicals used were the highest grade available. Glucose, fructose, mannose, agar powder, KH_2_PO_4_, (NH_4_)_2_SO_4_ and MgSO_4_ · 7H_2_O were purchased from Nacalai Tesque, Inc. (Kyoto, Japan). Malt extract and yeast extract were purchased from Becton, Dickinson and Company (BD, Franklin Lakes, NJ). Starch from corn, crystalline cellulose Sigmacell type 20 and xylan from birchwood were purchased from Sigma-Aldrich (St. Louis, MO).

### Microorganism and culture conditions

The *T. versicolor* strain used in this study, KT9427, was isolated from fruiting bodies on decaying wood in Tottori Prefecture, Japan. Strain KT9427 was determined to be dikaryotic because the formation of clamp connections, which are involved in the distribution of nuclei during mitosis, was observed microscopically. Fungal cultures were maintained on MYG medium agar slants, consisting of 10 g/l malt extract, 4 g/l yeast extract, 4 g/l glucose and 15 g/l agar powder, incubated at 28°C and then stored at 4°C. Subcultures were routinely made every six months. For fermentation experiments, *T. versicolor* strain KT9427 was grown on a MYG agar plate at 28°C for 7 days and three 0.5-cm^2^ pieces of the resulting mycelial mat were inoculated into a 500-ml Erlenmeyer flask containing 50 ml MYG liquid medium. After incubation for 7 days at 28°C, cells were harvested and transferred aseptically to a 500-ml Erlenmeyer flask containing 50 ml T1 medium (20 g/l xylose, 1 g/l yeast extract, 10 g/l KH_2_PO_4_, 2 g/l (NH_4_)_2_SO_4_ and 0.5 g/l MgSO_4_ · 7H_2_O, pH 4.7 for fermentation at 28°C without shaking. Xylose in T1 medium was replaced by other carbon sources at the identical concentration when the fermentation characteristics of *T. versicolor* towards various sugars and lignocellulosic materials were evaluated. Oxygen-limited and anaerobic conditions for the static cultures were generated by the absence or presence, respectively, of nitrogen gas in the headspace of the culture flasks, which were capped with silicon rubber plugs (As One Co., Ltd., Osaka, Japan). For aerobic conditions, the cultures were grown at 28°C in 500-ml Erlenmeyer flasks containing 50 ml T1 medium and capped with Silicosen stoppers (Shin-Etsu Polymer Co., Ltd., Tokyo, Japan), which is an air-permeable sponge plug made from silicone resin, with shaking at 180 rpm on a rotary shaker BR-33FL (Taitec Co., Ltd., Saitama, Japan).

### Determination of strain identity

Identification of strain KT9427 was based on standard morphological and biochemical analyses that were conducted at TechnoSuruga Laboratory Co., Ltd. (Shizuoka, Japan). The strain identity was confirmed by sequencing of the 28S rDNA D1/D2 domain. DNA was extracted from mycelia using a DNeasy Plant Mini kit (Qiagen, Hilden, Germany) according to the manufacturer’s instructions, and PCR was performed using puRe*Taq* Ready-To-Go PCR Beads (GE Healthcare, Buckinghamshire, England). Partial sequences were amplified with two primers (NL1 and NL4) targeting the D1/D2 domain (O’Donnell 1993). DNA sequencing was performed using an ABI Prism 3130x1 Genetic Analyzer System (Applied Biosystems, Foster City, CA). DNA sequences were compared with those of the GenBank database using the BLAST program (Altschul et al. 1997), and multiple alignments were conducted using ClustalW (Thompson et al. 1994). Most *T. versicolor* strains deposited in the GenBank/EMBL/DDBJ databases, including those under accession numbers AM269878, AY333793, AY684159, DQ208416 and DQ208417, had 100% sequence identity to 28S rDNA of strain KT9427. According to the morphological, biochemical and phylogenetic analyses, strain KT9427 was identified as *T. versicolor*. The 28S rDNA sequence from *T. versicolor* strain KT9427 determined in this study has been deposited in the GenBank/EMBL/DDBJ databases under accession number AB604944.

### Analytical methods

At periodic intervals during the fermentation experiments, 1-ml samples were collected in a clean bench or anaerobic chamber, as required based on the culture condition, centrifuged for 10 min at 15,000 × *g* in an Eppendorf benchtop centrifuge, and then filtered through a 0.22-μm membrane filter (Millex-GP; Millipore Corp., Billerica, MA). Ethanol, xylose, xylitol, glucose, mannose, fructose, cellobiose and maltose concentrations in the culture filtrates were determined by high-performance liquid chromatography (HPLC; Shimadzu Co., Ltd., Kyoto, Japan) equipped with a refractive index detector RID-10A and a Shodex KS-801 or SP0810 column (8.0 mm × 300 mm; Showa Denko Co., Ltd., Tokyo, Japan). The HPLC system was operated at 80°C using a distilled water mobile phase at a flow rate of 0.6 ml/min. The composition of wheat bran and rice straw were analyzed using the NREL Laboratory Analytical Procedures (Sluiter et al. 2008). Reducing sugars liberated during the fermentation were measured using the dinitrosalicylic acid (DNS) method (Miller 1959). The concentrations of starch and glucose in the culture filtrates during fermentation on corn starch and wheat bran were determined using an F-kit for starch (Roche Diagnostics, Manheim, Germany) and Glucose C2 test kit (Wako Pure Chemical Industries, Ltd.), respectively. The theoretical yield of ethanol was defined as 0.51 g ethanol per 1 g glucose (2 mol of ethanol per 1 mol of glucose) or 0.51 g ethanol per 1 g xylose (1.67 mol of ethanol per 1 mol of xylose).

## Results

### Isolation of xylose-fermenting strain

**Figure 1:**
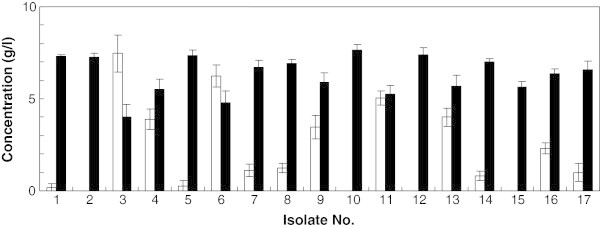
**Ethanol production from xylose by** ***T. versicolor*** **isolates after 120 h of fermentation.** Bars indicate ethanol (black) and residual xylose (white) concentrations.

Seventeen *T. versicolor* isolates collected from many locations in Japan were tested for xylose fermentation under oxygen-limited conditions. Although the fermentation performance varied among strains, all of the isolates produced ethanol with yields of 0.32 - 0.40 g ethanol g per 1 g xylose consumed (Figure 1), indicating that these strains have the capability to ferment xylose to ethanol. Among the examined strains, isolate No. 10 exhibited the highest xylose consumption rate and yield of ethanol. Therefore, this strain, designated KT9427, was selected for further experimental analyses.

### Effect of aeration on xylose fermentation

**Figure 2:**
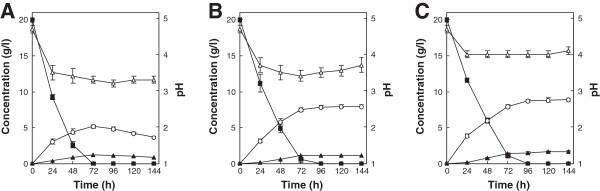
**Ethanol production from xylose by** ***T. versicolor*** **KT9427 under aerobic (A), oxygen-limited (B) and anaerobic conditions (C).** Symbols represent xylose (filled squares), xylitol (filled triangles), ethanol (open circles) and pH (open triangles). Error bars indicate standard deviations of the means from three independent experiments.

The consumption and fermentation of xylose by *T. versicolor* KT9427 were first investigated under three different aeration conditions (Figure 2). When strain KT9427 was cultured under aerobic, oxygen-limited and anaerobic conditions, xylose was completely consumed after 72, 96 and 96 h of fermentation, respectively, with maximum ethanol concentrations of 5.2, 7.9 and 8.8 g/liter, respectively. These results indicated that the maximum ethanol yield and volumetric ethanol productivity during xylose fermentation by *T. versicolor* occurred under anaerobic conditions. Thus, the fermentation ability of strain KT9427 was further examined under anaerobic conditions.

### Effect of initial pH on xylose fermentation

**Figure 3:**
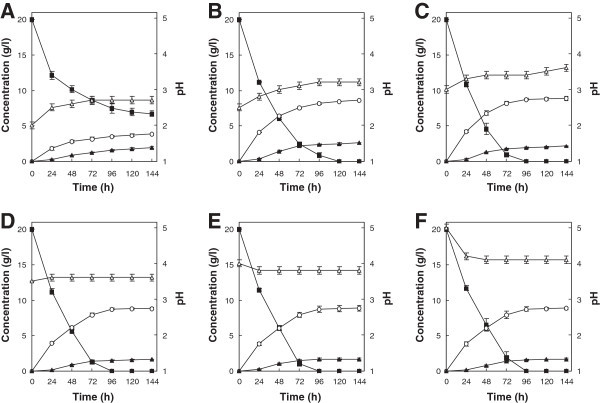
**Ethanol production from xylose by** ***T. versicolor*** **KT9427 at an initial pH of 2 (A), 2.5 (B), 3 (C), 3.5 (D), 4 (E) and 5 (F).** Symbols represent xylose (filled squares), xylitol (filled triangles), ethanol (open circles) and pH (open triangles). Error bars indicate standard deviations of the means from three independent experiments.

The xylose fermentation ability of *T. versicolor* KT9427 cultured in T1 medium at initial pHs ranging from 2 to 5 was investigated (Figure 3). A maximum ethanol concentration of 8.8 g/l was reached after 96 h of fermentation at pHs of 3 and higher. Although xylose consumption was slightly delayed at pH 2.5, requiring 120 h to be completely depleted, the final ethanol concentration was similar to those achieved at the higher pHs. However, at an initial pH 2.0, xylose consumption and ethanol production were dramatically reduced.

### Production of ethanol from hexose sugars

**Figure 4:**
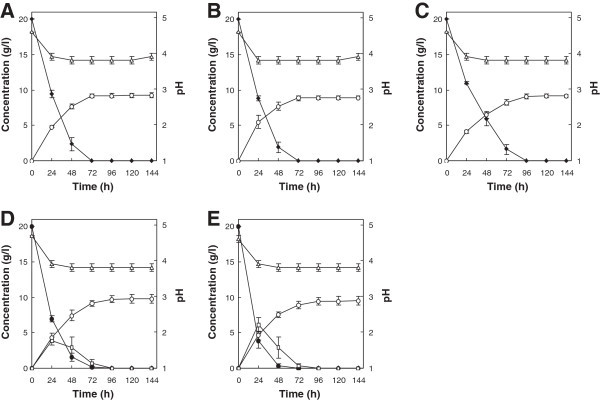
**Ethanol production from glucose (A), mannose (B), fructose (C), cellobiose (D) and maltose (E) by** ***T. versicolor*** **KT9427.** Symbols represent hexose monosaccharide (filled diamonds), disaccharide (filled circles), liberated glucose (open squares), ethanol (open circles) and pH (open triangles). Error bars indicate standard deviations of the means from three independent experiments.

The fermentation performance of strain KT9427 was next investigated in T1 medium containing one of several different hexose sugars as the sole carbon source at an initial concentration of 20 g/l. The monosaccharides glucose, mannose and fructose were completely consumed within 72, 72 and 96 h, respectively (Figure 4A, B and C). Maximum ethanol concentrations of 9.2, 8.9 and 9.1 g/l, which corresponded to ethanol yields of 0.46, 0.45 and 0.46 g ethanol per 1 g sugar consumed, respectively, were reached in the glucose-, mannose- and fructose-supplemented media, respectively. When *T. versicolor* was cultured in T1 medium containing 20 g/l of either cellobiose or maltose as the sole carbon source, both types of disaccharides were completely consumed within 96 h, and glucose was liberated in the culture medium during the first 72 h of fermentation (Figure 4D and E). The maximum ethanol concentrations in the respective cultures were 9.8 and 9.5 g/l, which corresponded to yields of 0.49 and 0.48 g ethanol per 1 g sugar consumed, respectively.

### Direct ethanol production from starch, cellulose, xylan, wheat bran and rice straw

**Figure 5:**
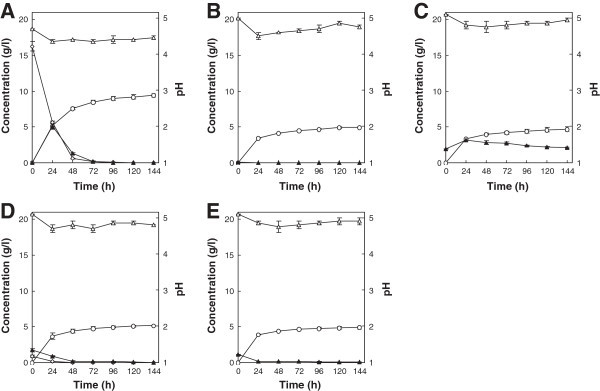
**Ethanol production from starch (A), cellulose (B), xylan (C), wheat bran (D) and rice straw (E) by** ***T. versicolor*** **KT9427.** Symbols represent starch (open diamonds), reducing sugar (filled triangles), ethanol (open circles) and pH (open triangles). Error bars indicate standard deviations of the means from three independent experiments.

As it was demonstrated that *T. versicolor* could assimilate and effectively ferment a broad spectrum of carbon sources, the direct fermentation performance of this basidiomycete on starch, cellulose, xylan, wheat bran and rice straw, was also evaluated. Because large-scale, cost-effective CBP processes for bioethanol production will likely require the use of raw, untreated lignocellulosic starting materials, the five examined carbon sources were not pre-treated by hydrolysis with acid, alkali or enzymes, although the commercially manufactured cellulose and xylan had likely been purified by various pre-treatment processes. When *T. versicolor* KT9427 was cultured in T1 medium supplemented with 20 g/l corn starch without pH adjustment, a maximum ethanol concentration of 9.8 g/l was reached after 96 h of fermentation (Figure 5A). A small amount of reducing sugar was detected in the culture medium during the first 72 h of fermentation and was attributed to the rapid uptake and conversion of starch. The direct production of ethanol by *T. versicolor* from the other four carbon sources was also confirmed. Using T1 medium supplemented with 20 g/l crystalline cellulose (Sigmacell type 20) or birchwood xylan without pH adjustment, maximum ethanol concentrations of 4.7 and 4.4 g/l, respectively, were achieved after 96 h of fermentation (Figure 5B and C). Finally, when cells were cultured in T1 medium supplemented with either 20 g/l wheat bran or rice straw without pH adjustment, maximum ethanol concentrations of 5.0 and 4.8 g/l, respectively, were observed after 96 h of fermentation (Figure 5D and E).

## Discussion

With the exception of several fungal species, most alcohol-fermenting fungi characterized to date are incapable of converting xylose into ethanol. Here, we demonstrated that *T. versicolor* is able to efficiently assimilate and convert xylose into ethanol under both oxygen-limited and anaerobic conditions. *T. versicolor* has also been observed to produce ethanol from xylose under aerobic conditions with shaking. A similar property has also been found in zygomycete strains (Millati et al. 2005). Generally, xylose-fermenting yeasts require oxygen for xylose metabolism, but exhibit maximum ethanol yields under oxygen-limited conditions (Ligthelm et al. 1988). However, du Preez et al. (1984) reported that aeration enhanced ethanol productivity by *Candida shehatae* and *Pachysolen tannophilus*, and no ethanol formation was detected under anaerobic conditions by the zygomycete *Mucor indicus* or the representative xylose-fermenting yeast *P. stipitis* (Karimi et al. 2006). In contrast, here, reduced aeration resulted in increased ethanol production from xylose by *T. versicolor*, a trend that is in agreement with that observed for the fermentation of hexose sugars. Therefore, our findings suggest that *T. versicolor* possesses a different xylose fermentation mechanism from those of yeasts and zygomycetes characterized previously, and that anaerobic conditions yield the highest ethanol productivity. During the fermentation of xylose by *T. versicolor*, xylitol accumulated at levels that were influenced by the degree of oxygen limitation. This result suggests that the conversion of xylose to ethanol in *T. versicolor* is also controlled by the rate of xylose metabolism and may be closely influenced by the redox imbalance between the conversion of xylose to xylitol and xylitol to xylulose.

When the initial pH of the culture medium was lowered from 5.0 to 3.0, xylose fermentation by *T. versicolor* was not significantly affected. Furthermore, although an initial medium pH of 2.5 resulted in a slightly decreased rate of xylose consumption, the ethanol yield was not markedly affected. As the pH of the medium dropped, however, xylitol accumulation tended to increase. This response may be due to inhibition of the pentose phosphate pathway, which is one pathway used by *T. versicolor* for xylose conversion at low pH, without corresponding inhibition of the pathway responsible for the conversion of pyruvate to ethanol. Although a further drop in pH to 2.0 dramatically inhibited xylose consumption during fermentation, a relatively good ethanol yield (0.32 g ethanol per 1 g of xylose consumed) was obtained. Although *P. tannophilus* is also able to ferment xylose at pH 2.5, the ethanol yield (0.20 g/g xylose consumed) under this condition is extremely low (Slininger et al. 1982), and most xylose-fermenting yeasts show optimal activity in the region of pH 4.0 - 5.5 (du Preez et al. 1986; Slininger et al. 1990). To our knowledge, efficient ethanol production at low medium pH has not been reported in any other xylose-fermenting microorganism to date. Our results indicate that the pH limit for efficient xylose fermentation by *T. versicolor* is approximately pH 2.5, indicating that pH control may not be essential during large-scale fermentations.

*T. versicolor* exhibited relatively efficient ethanol production from not only xylose, but also from various hexose sugars. The examined hexose sugars were consumed within 96 h and converted to ethanol with maximum yields of 0.45 - 0.49 g ethanol/g sugar, which were superior to the yields obtained for xylose fermentation under similar conditions. Notably, the fermentation performance of *T. versicolor* reported here is the highest among the main species of white rot basidiomycetes with fermentability that have been isolated to date (Kenealy and Dietrich 2004; Mizuno et al. 2009; Okamoto et al. 2010, 2011). Thus, *T. versicolor* represents a suitable candidate for use in ethanol production from the hydrolyzate of lignocellulosic biomass, which typically contains glucose, mannose and xylose, as this fungus is able to assimilate and ferment a wide spectrum of sugars with good ethanol yield.

In our fermentation experiments, *T. versicolor* produced ethanol directly from starch, which was rapidly decomposed and assimilated by cells. The ethanol yield (0.49 g ethanol per 1 g starch) of this naturally occurring basidiomycete was superior to that of a recombinant starch-utilizing strain of *S. cerevisiae*, which was previously reported to produce 61.8 g/l ethanol from 200 g starch after 72 h of fermentation (Shigechi et al. 2004), corresponding to a yield of 0.31 g ethanol per 1 g starch. The present findings suggest that *T. versicolor* secretes functional starch-degrading enzymes that are closely linked to the fermentation of ethanol.

*T. versicolor* was capable of directly fermenting crystalline cellulose to ethanol with relatively good yield compared to those of previously characterized ascomycete *Aspergillus* species and zygomycete *Rhizopus* species (Skory et al. 1997). Most previous lignocellulosic bioethanol studies have examined the simultaneous saccharification and fermentation (SSF) of crystalline cellulose, such as Avicel and Sigmacell, with natural yeasts or recombinant bacteria and achieved high ethanol yields through the addition of external cellulase (Ingram and Doran 1995; Spindler et al. 1998). Considering that *T. versicolor* does not require the hydrolysis pre-treatment of cellulose polymers for their subsequent saccharification, this basidiomycete is a promising candidate for ethanol production from cellulosic substrates, including birchwood xylan, which was directly converted into ethanol. Although a recombinant strain of xylan-utilizing *Klebsiella oxytoca* was reported to produce 7.8 g/l ethanol from 40 g birchwood xylan after 60 h of fermentation (Burchhardt and Ingram 1992), the conversion process consisted of two steps, hydrolysis and fermentation, whereas that of *T. versicolor* occurs in a single fermentation step. A recombinant xylan-utilizing strain of *S. cerevisiae* was also reported to produce 7.1 g/l ethanol from 100 g non-pretreated birchwood xylan after 62 h of fermentation (Katahira et al. 2004); however, this yield is lower than that of the genetically unmodified strain of *T. versicolor* characterized here, which produced 4.0 g/l ethanol from 20 g/l non-pretreated birchwood xylan after 48 h of fermentation. Although hydrolysis products, such as glucose and xylose, were not detected at significant levels in the culture medium during the fermentation of either cellulose or xylan, both of these polymers might be accessible and hydrolyzed into monomer sugars or disaccharides. Based on the analysis of culture medium using the DNS method, it appears that *T. versicolor* rapidly assimilates sugars liberated from polysaccharides in the medium after hydrolysis. To our knowledge, little evidence exists for direct ethanol production from microcrystalline cellulose or xylan by basidiomycetes. Thus, our present findings indicate that *T. versicolor* secretes highly active enzymes that are involved in polymer hydrolysis and ethanol production, and make this basidiomycete a suitable candidate for CBP.

To further explore the fermentation properties of *T. versicolor*, fermentation experiments with wheat bran and rice straw as representative lignocellulosic starting materials were conducted. The monosaccharides glucose, xylose and galactose present in the wheat bran, which was composed of 35.7% (wt/wt) glucose (mainly in the form of starch), 16.9% (wt/wt) xylose, 9.7% (wt/wt) arabinose, 1.0% (wt/wt) galactose, 7.8% (wt/wt) lignin, 17.5% (wt/wt) crude protein, 6.8% (wt/wt) crude fat and 4.6% (wt/wt) ash, are considered to be the main fermentable sugars (total 10.7 g/l with 100% recovery). *T. versicolor* was estimated to convert wheat bran to ethanol at 92% of the theoretical yield, indicating that this basidiomycete is able to hydrolyze and ferment the hemicellulose fraction in lignocellulosic biomass. In the case of rice straw, which was composed of 29.9% (wt/wt) glucose, 15.6% (wt/wt) xylose, 3.2% (wt/wt) arabinose, 3.5% (wt/wt) mannose, 2.3% (wt/wt) galactose, 19.7% (wt/wt) lignin and 17.8% (wt/wt) ash, the fermentable sugars glucose, xylose, galactose and mannose (total 10.3 g/l with 100% recovery) served as readily available carbon sources. Despite differences in the linkage of glucose units between the starch found in wheat bran and the cellulose present in rice straw, *T. versicolor* was also able to convert rice straw to ethanol at 91% of the theoretical yield. Using similar approaches, direct ethanol production from lignocellulosic material by *N. crassa* and *F. oxysporum* previously resulted in yields of 0.074 and 0.109 g ethanol per 1 g alkali-pretreated brewer’s spent grain, respectively (Xiros et al. 2008; Xiros and Christakopoulos 2009). Even after taking into account the different forms and sugar composition of the source materials, these ethanol yields are markedly lower than the yields of 0.248 g ethanol per 1 g non-pretreated wheat bran and 0.239 g ethanol per 1 g non-pretreated rice straw observed in the present study. The ethanol yields of *T. versicolor* are also superior to those from the fermentation of lignocellulosic biomass by *T. hirsuta* (Okamoto et al. 2011), a result that is likely due to differences in the fermentability of xylose between these two species.

To our knowledge, this is the first report to describe the efficient fermentation of xylose and xylose-containing lignocellulosic biomass by a white rot basidiomycete. *T. versicolor* was capable of assimilating a broad spectrum of carbon sources and displayed a favorable ability to ferment xylose, which is abundant in agricultural residues such as wheat bran and rice straw. These properties indicate that *T. versicolor* is a promising microorganism for the production of bioethanol from lignocellulosic biomass. In addition, the use of this basidiomycete for industrial bioethanol production would have clear advantages over *S. cerevisiae* and *P. stipitis*, as *T. versicolor* is able to directly convert starch, cellulose, xylan, wheat bran and rice straw into ethanol without the need for costly pre-treatment processing. Optimization of the fermentation conditions is expected to further enhance ethanol productivity and lower xylitol accumulation by *T. versicolor*.

In conclusion, *T. versicolor* appears to possess well-balanced conversion systems for the direct production of ethanol from various starting materials without the need for genetic engineering, and is potentially an ideal microbe for CBP of bioethanol and lignocellulose-based biorefinery processes.
